# Supplementary material for: Ecology and Function of the Transmissible Locus of Stress Tolerance in Escherichia coli and Plant-Associated Enterobacteriaceae
Source: mSystems. 2021 Aug 17;6(4):e00378-21. doi: 10.1128/mSystems.00378-21 (PMC8407380; doi:10.1128/mSystems.00378-21)
Supplement: TABLE S1 [file msystems.00378-21-st001.pdf]

Table S1. Occurrence and prevalence of the tLST in strains of *Klebsiella pneumononiae* and *Cronobacter sakazakii* of different origin.

Species	# of genomes	% tLST positive genomes
Cronobacter sakazakii	355	15
Clinical isolates	101	8
Environmental isolates	81	8
Klebsiella pneumoniae	4162	7
Clinical isolates	250	4
Environmental isolates	360	2
