# Supplementary material for: Ecology and Function of the Transmissible Locus of Stress Tolerance in Escherichia coli and Plant-Associated Enterobacteriaceae
Source: mSystems. 2021 Aug 17;6(4):e00378-21. doi: 10.1128/mSystems.00378-21 (PMC8407380; doi:10.1128/mSystems.00378-21)
Supplement: FIG S1 [file msystems.00378-21-sf001.pdf]

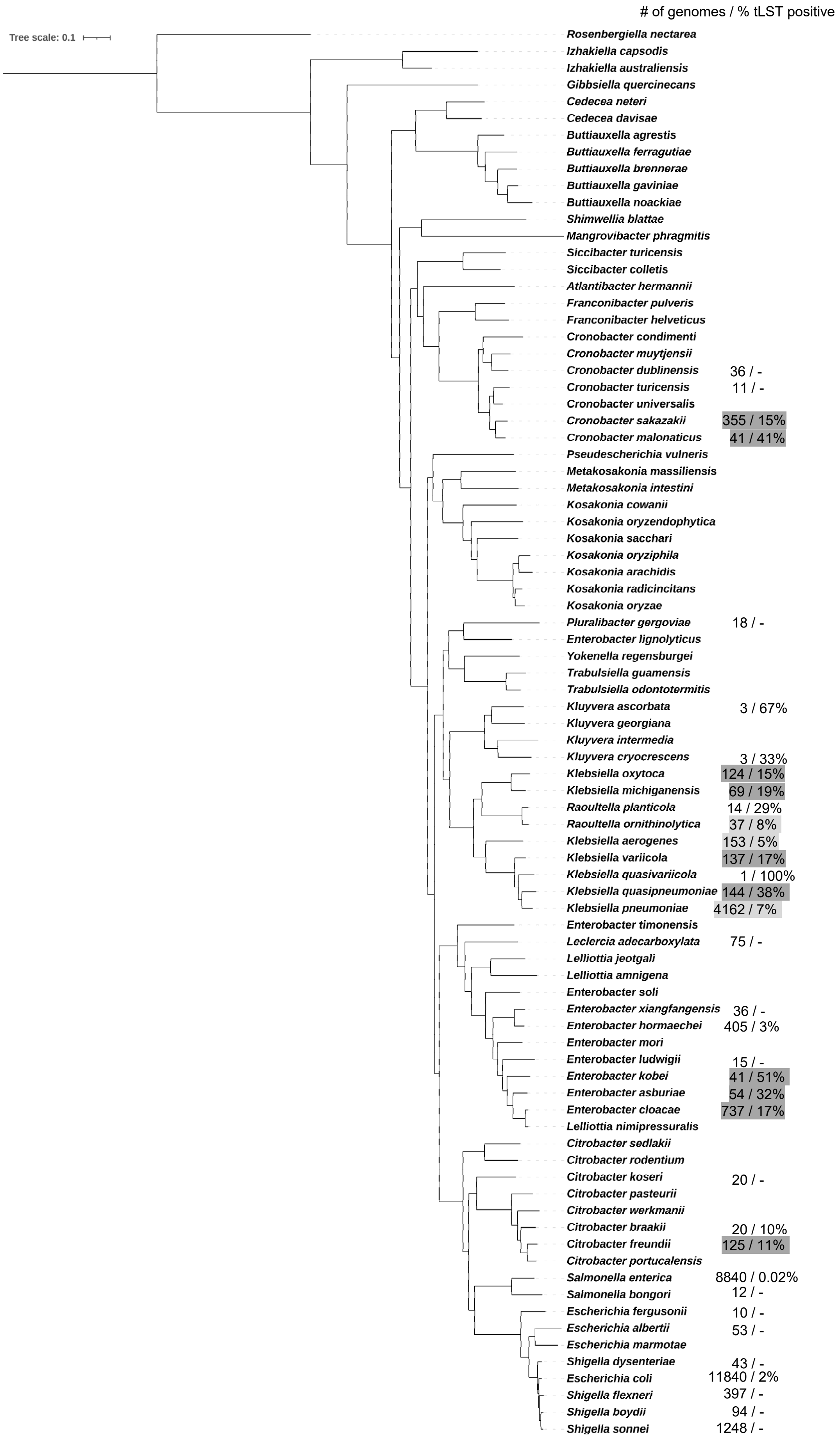

Tree scale: 0.1
# of genomes / % tLST positive
Rosenbergiella nectarea
Izhakiella capsodis
Izhakiella australiensis
Gibbsiella quercinecans
Cedecea neteri
Cedecea davisae
Buttiauxella agrestis
Buttiauxella ferragutiae
Buttiauxella brennerae
Buttiauxella gaviniae
Buttiauxella noackiae
Shimwellia blattae
Mangrovibacter phragmitis
Siccibacter turicensis
Siccibacter colletis
Atlantibacter hermannii
Franconibacter pulveris
Franconibacter helveticus
Cronobacter condimenti
Cronobacter muytjensii
Cronobacter dublinensis 36 / -
Cronobacter turicensis 11 / -
Cronobacter universalis
Cronobacter sakazakii 355 / 15%
Cronobacter malonaticus 41 / 41%
Pseudescherichia vulneris
Metakosakonia massiliensis
Metakosakonia intestini
Kosakonia cowanii
Kosakonia oryzendophytica
Kosakonia sacchari
Kosakonia oryziphila
Kosakonia arachidis
Kosakonia radicincitans
Kosakonia oryzae
Pluralibacter gergoviae 18 / -
Enterobacter lignolyticus
Yokenella regensburgei
Trabulsiella guamensis
Trabulsiella odontotermitis
Kluyvera ascorbata 3 / 67%
Kluyvera georgiana
Kluyvera intermedia
Kluyvera cryocrescens 3 / 33%
Klebsiella oxytoca 124 / 15%
Klebsiella michiganensis 69 / 19%
Raoultella planticola 14 / 29%
Raoultella ornithinolytica 37 / 8%
Klebsiella aerogenes 153 / 5%
Klebsiella variicola 137 / 17%
Klebsiella quasivariicola 1 / 100%
Klebsiella quasipneumoniae 144 / 38%
Klebsiella pneumoniae 4162 / 7%
Enterobacter timonensis
Leclercia adecarboxylata 75 / -
Lelliottia jeotgali
Lelliottia amnigena
Enterobacter soli
Enterobacter xiangfangensis 36 / -
Enterobacter hormaechei 405 / 3%
Enterobacter mori
Enterobacter ludwigii 15 / -
Enterobacter kobei 41 / 51%
Enterobacter asburiae 54 / 32%
Enterobacter cloacae 737 / 17%
Lelliottia nimipressuralis
Citrobacter sedlakii
Citrobacter rodentium
Citrobacter koseri 20 / -
Citrobacter pasteurii
Citrobacter werkmanii
Citrobacter braakii 20 / 10%
Citrobacter freundii 125 / 11%
Citrobacter portucalensis
Salmonella enterica 8840 / 0.02%
Salmonella bongori 12 / -
Escherichia fergusonii 10 / -
Escherichia albertii 53 / -
Escherichia marmotae
Shigella dysenteriae 43 / -
Escherichia coli 11840 / 2%
Shigella flexneri 397 / -
Shigella boydii 94 / -
Shigella sonnei 1248 / -

Figure S1. Phylogenetic tree of representative genomes of type strains of the *Enterobacteriaceae* (left) with indication of the number of genomes and the percent of genomes that are positive for one of the two tLST variants. The number of genomes is indicated only for those species that were represented by 10 or more genomes in the genome database (Table S2); gray shading identifies those species that are represented by more than 30 genomes and include more than 5% tLST positive genomes; dark gray shading identifies those species that are represented by more than 30 genomes and include more than 15% tLST positive genomes. The core genome phylogenetic tree was constructed as described (4). In brief, genomes are labelled as "ncbi_type_material" in the family *Enterobacteriaceae* were retrieved in June 2021. Core genes were identified with Roary (5) with 80% identity. Core genes (289) were used to construct maximum likelihood (ML) phylogenetic tree (6).
