# Supplementary material for: Ecology and Function of the Transmissible Locus of Stress Tolerance in Escherichia coli and Plant-Associated Enterobacteriaceae
Source: mSystems. 2021 Aug 17;6(4):e00378-21. doi: 10.1128/mSystems.00378-21 (PMC8407380; doi:10.1128/mSystems.00378-21)
Supplement: FIG S2 [file msystems.00378-21-sf002.pdf]

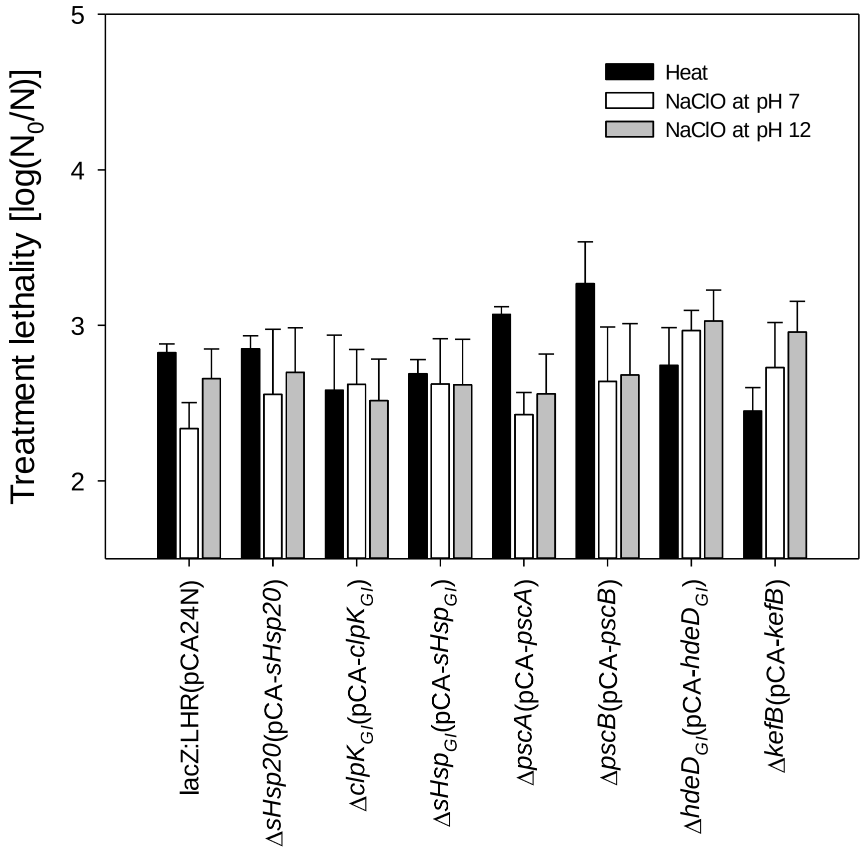

Figure S2. Lethality of heat and chlorine treatments to cultures of complemented *E. coli* MG1655 *lacZ::*tLST with single deletion. The empty vector pCA24N was transformed to *E. coli* MG1655 *lacZ::*tLST as negative control. The treatment lethality was expressed as the log-transformed ratio of cell counts before treatment (N_0) over the cell counts after treatment (N). Black bars represent cells treated after 63 °C for 5 min; white bars represent cells treated with 50 mM NaClO for 5 min at pH 7; grey bars represent cells treated with 8 mM NaClO for 5 min at pH 12. Values for different strains within a treatment are not significantly different (P>0.05). Data are shown as means ± standard deviation of three independent experiments.
