# Supplementary material for: Ecology and Function of the Transmissible Locus of Stress Tolerance in Escherichia coli and Plant-Associated Enterobacteriaceae
Source: mSystems. 2021 Aug 17;6(4):e00378-21. doi: 10.1128/mSystems.00378-21 (PMC8407380; doi:10.1128/mSystems.00378-21)
Supplement: FIG S3 [file msystems.00378-21-sf003.pdf]

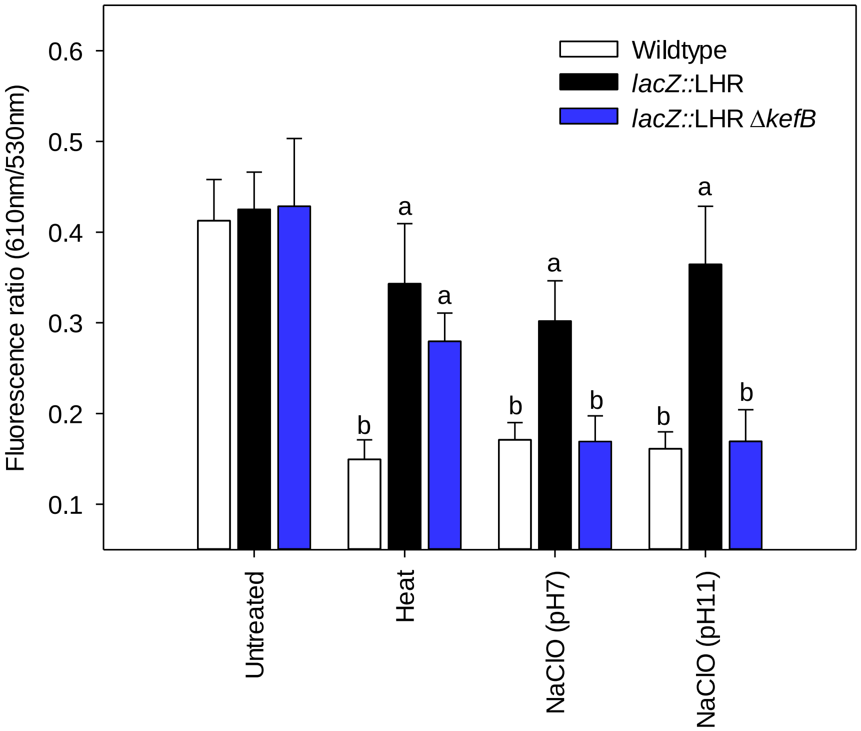

Figure S3. Quantification of the membrane potential in *E. coli* MG1655, *E. coli* MG1655 *lacZ::*tLST and *E. coli* MG1655 *lacZ::*tLST Δ*kefB*. The ratio of the fluorescence intensity at emission wavelengths of 610 and 530 nm was calculated to indicate the membrane potential. Cells were stained with 30 µM DiOC2(3) and then were treated with 60 °C, 25 mM NaClO at pH 7 or 4 mM NaClO at pH 11 for 5 min to cause reduction of cell counts from 0.5 to 5 logCFU/mL. Values for different strains that do not have a common superscript are significantly different (P<0.05). Data are shown as means ± standard deviation of at least three independent experiments.
