# Supplementary material for: Ecology and Function of the Transmissible Locus of Stress Tolerance in Escherichia coli and Plant-Associated Enterobacteriaceae
Source: mSystems. 2021 Aug 17;6(4):e00378-21. doi: 10.1128/mSystems.00378-21 (PMC8407380; doi:10.1128/mSystems.00378-21)
Supplement: TABLE S3 [file msystems.00378-21-st003.pdf]

Table S3. Strains used in Figure 3 and their origin or source of isolation.

Strains	tLST variant	Source	Latitude	Longitude	Reference
E.coli FUA10289	tLST1	North Saskatchewan River	53.5317	-113.5119	This study
E.coli FUA10290	-[a]	North Saskatchewan River	53.5317	-113.5119	This study
E.coli FUA10291	tLST1	Hawrelak Park	53.5272	-113.5490	This study
E.coli FUA10292	tLST1	Unknown Lake	52.1542	-117.0097	This study
E.coli FUA10293	-	Unknown Lake	52.1542	-117.0097	This study
E.coli FUA10296	tLST1	Chain Lakes	50.2030	-114.1926	This study
E. coli FUA10297	tLST1, tLSTa	Pond beside highway	50.3426	-113.7657	This study
K. pneumoniae FUA10298	tLSTa	Sheep river	50.7129	-113.8813	This study
K. pneumoniae FUA10329	-	Clinical isolate[b]			
K. pneumoniae FUA10330	-	Clinical isolate[b]			
K. pneumoniae FUA1427	tLST1	Human intestine			(1, 2)
K. oxytoca FUA10326	-	Clinical isolate[b]			
K. oxytoca FUA10327	-	Clinical isolate[b]			
K. oxytoca FUA10328	-	Clinical isolate[b]			
K. oxytoca FUA1261	-	Rat mesenteric lymph nodes			(3)
K. oxytoca FUA1266	-	Rat mesenteric lymph nodes			(3)
K. oxytoca FUA1271	-	Rat mesenteric lymph nodes			(3)

[a] "-"indicates tLST-negative

[b] The clinical isolates were obtained by Michael E. Stiles prior to 1985 and the exact source of isolation is unknown.
