# Supplementary material for: Ecology and Function of the Transmissible Locus of Stress Tolerance in Escherichia coli and Plant-Associated Enterobacteriaceae
Source: mSystems. 2021 Aug 17;6(4):e00378-21. doi: 10.1128/mSystems.00378-21 (PMC8407380; doi:10.1128/mSystems.00378-21)
Supplement: TABLE S4 [file msystems.00378-21-st004.pdf]

Table S4. Primers and probes used in this study.

Assay	Primer/probe name	Primer/probe sequence (5’-3’)	Tm (°C)
tLST versions	tLST1-F1-F	GTCGTCTACAAGCGTGATCC	60
	tLST1-F1-R	CAGAGGATCAGGTGAACAGAC	
	tLST1-F2-F	CGATATTTTCGCTGGCAGTCAG	
	tLST1-F2-R	CCTTCAGCAGGTCAAATGGAAA	
	tLST1-F3-F	AGCTCATCTCCATCGTCTTCAC	
	tLST1-F3-R	TCTTGACCTGGATGATGCTGTC	
	tLST2-F1-F	CGTTCAACCCGACATTGATTCC	
	tLST2-F1-R	ACGAAAGTGCAAATCGTGACAG	
	tLST2-F2-F	GTGCAGCTGCCACTATCTATCT	
	tLST2-F2-R	CGATGACGCGCATATATGTGTC	
	tLST2-F3-F	TCAATGATACCTATGGGCACGG	
	tLST2-F3-R	TTCCAACAACACAGCGAATTCC	
MG1655 *lacZ::*tLST construction	sgRNA-lacZ-F	GGCCAGTGAATCCGTAATCAGTTTTAGAGCTAGAAATAGCAAG	62
	sgRNA-lacZ-R	TGATTACGGATTCACTGGCCGTGCTCAGTATCTCTATCACTGA	
	Targeting sequence	GGCCAGTGAATCCGTAATCA	
	tLST-16-F	CGGTATCGCCGTCGACGACG	
	lacZ-upstream	GCTGTTGCCCGTCTCACTGG	
	tLST-2-R	GCCGGAATTTCCCCGTGTGC	
	lacZ-downstream	GGACGACGACAGTATCGGCC	
Cm^R cassette	*orf1*-P2	TTGGCCGGAACCGCAAAGCGACGCGATTGGAGAGGGTGAGATGGGAATTAGCCATGGTCC	65
	orf1-P1	GTCCATGTCATACCTCCAGAAAAATTGGAAAACGAGTTACGTGTAGGCTGGAGCTGCTTC	

sHsp20-P2	GCTTGAGTAACTCGTTTTCCAATTTTTCTGGAGGTATGACATGGGAATTAGCCATGGTCC
sHsp20-P1	CTCGGGACGGGTTTTTCTTGTGACGAACGAGCCGGCGACGGTGTAGGCTGGAGCTGCTTC
clpK$_{GI}$-P2	GAGGTGCTGGCCCGGCGCAAGAAGAACAACCCGGTGCTGAATGGGAATTAGCCATGGTCC
clpK$_{GI}$-P1	GCCAGGCCCTTGGGGTCGGACACGGCAGCCTCGCCCGCCAGTGTAGGCTGGAGCTGCTTC
sHsp$_{GI}$-P2	GCGTCATCCAATTCGTCGGCATTGTTCAAGGAGAACCGATATGGGAATTAGCCATGGTCC
sHsp$_{GI}$-P1	TGGCTGTTTGATATTCATGATCTTGGGCTCCTGATTGAAGGTGTAGGCTGGAGCTGCTTC
pscA$_{GI}$-P2	GAAATCTCAGTCAACTAACTTCAATCAGGAGCCCAAGATCATGGGAATTAGCCATGGTCC
pscA$_{GI}$-P1	CGCGAGACTGACCGACAGAGCAGTCAGCCCCTTCATCACCGTGTAGGCTGGAGCTGCTTC
pscB-P2	GATTCGCCTATTTTCACTATCTCATACGAGGCATATCACCATGGGAATTAGCCATGGTCC
pscB-P1	AGGGGCGAGACGCGCCCCTCTTACGCCCTATCCAGCGGTTGTGTAGGCTGGAGCTGCTTC
hdeD$_{GI}$-P2	GTTTGCTTTTTCACCCCATTAGATCTTTAGGAGATATAGCATGGGAATTAGCCATGGTCC
hdeD$_{GI}$-P1	CGAGGATGCTCATATCCATAGATTTTGGTTTGGCACGCTCGTGTAGGCTGGAGCTGCTTC
orf11-P2	GATTCATGCCTCCATAGAAATGACATCGAAGGAGTCATACATGGGAATTAGCCATGGTCC
orf11-P1	GCGGTTGATGGACTGGCAGTGCGGGCAGACGAGGTGAAGAGTGTAGGCTGGAGCTGCTTC
trx$_{GI}$-P2	CGAAGCGAGGCAGCCTCACTGTGGTCCAAGGAGGCGAACCATGGGAATTAGCCATGGTCC
trx$_{GI}$-P1	CCAGCAGGATTAGTGTAGTGCCGAGCAAGCCCTGCATCGGGTGTAGGCTGGAGCTGCTTC
kefB-P2	CATCGTGCGCTGGACGTCGACGCAAGTGGGACGCTGACCGATGGGAATTAGCCATGGTCC
kefB-P1	TGGTCACGTAAGACCTGAAATGGGTTAAGGCGTGTTGATTGTGTAGGCTGGAGCTGCTTC
pseiE$_{GI}$-P2	GCCGTCTCGACAGCAGCCGTTTACCAGGGAGTTCATCGTCATGGGAATTAGCCATGGTCC
pseiE$_{GI}$-P1	GCGGTTGAGCCAGCGGTGTTGCAAGAGCGCCGTGCGCGGAGTGTAGGCTGGAGCTGCTTC
orf15-P2	CTGCGCAAGCGCGGCGACCGCGCCGCCGAGACCTCTGAGCATGGGAATTAGCCATGGTCC
orf15-P1	GGTCGAGATAGGCCATGAGACGTCTCCTGTTGATGGGCAAGTGTAGGCTGGAGCTGCTTC
orf16-P2	CGGGCTGTGGCGCTGATTGCCCATCAACAGGAGACGTCTCATGGGAATTAGCCATGGTCC
orf16-P1	CTTGGCTGCTTCGCTGTGCAGATCCATGACCCACTTTCCAGTGTAGGCTGGAGCTGCTTC
orf1-Up-F	TGCAAGGATTTCCTTGGCCG

Mutants check (Up/Down)	*orf1*-Down-R	CCTTAGAAGGTTGAAAAGGTTGAGG
	sHsp20-Up-F	AATTTTTCTGGAGGTATGAC
	sHsp20-Down-R	TGAGATTGGCTTCCACCC
	$clpK_{GI}$-Up-F	AAAACCCGTCCCGAGACG
	$clpK_{GI}$-Down-R	CCTTGGCCTCATCCCCCCCC
	$sHsp_{GI}$-Up-F	CGGCTCTGTTCAGTGCG
	$sHsp_{GI}$-Down-R	CAGAGGGTGCTGCAGCA
	$pscA_{GI}$-Up-F	CAACTAACTTCAATCAGGAGCCC
	$pscA_{GI}$-Down-R	GCTGCTTACCGCGGC
	pscB-Up-F	TCGCATTCAGCCCCAATAGA
	pscB-Down-R	TGGTGGGGTCATCCGC
	$hdeD_{GI}$-Up-F	CTCTCTTGGCTCAGGAGTGTT
	$hdeD_{GI}$-Down-R	AGCTCTCTGCCAAGGTTCAT
	orf11-Up-F	CTTCGCAAAACGCGCCT
	orf11-Down-R	CAAGGGCTGCTGGCAGC
	trx_{GI}-Up-F	AGGCGTTGATTGGTTGGC
	trx_{GI}-Down-R	GGGGCCCAGTACAACGC
	kefB-Up-F	TCGTGCGCTGGACGT
	kefB-Down-R	GCAGCGTGAAGACGATGG
	$pseiE_{GI}$-Up-F	GACACATCAGCGCAATGCG
	$pseiE_{GI}$-Down-R	GGCAAACAGCGCGAGC
	orf15-Up-F	TACTGGCTGCTGCGCA
	orf15-Down-R	ACCATGCCTCGATGGCG
	orf16-Up-F	TCCGGCAACGCAAGAGC
	orf16-Down-R	GCTGTGCAGATCCATGACC

Mutants check (absence)	*orf1*-check-F	GGTGATTTTCACGCTCGATG	60
	orf1-check-R	TCGGATGACTTCTGCTGTTC	
	sHsp20-check-F	TACAAGATTGCCCTGGAAGT	
	sHsp20-check-R	CTTGATCGAATCCTGGTTGG	
	$clpK_{GI}$-check-F	CCATTCTTATGTCGGTCCAGAG	
	$clpK_{GI}$-check-R	CCACCTTGCTGACCTGTT	
	$sHsp_{GI}$-check-F	TCCGGGAACTGGATGAATTG	
	$sHsp_{GI}$-check-R	AGATCCAGCTTGAGGAGGAA	
	$pscA_{GI}$-check-F	TCGGTAAAGAAAGCGGTCAAG	
	$pscA_{GI}$-check-R	CATCGGAAGGTTGTCGGTTT	
	pscB-check-F	CAAACACCGAATCCCAATGC	
	pscB-check-R	GCCTCATCGAGGACTTGTTT	
	$hdeD_{GI}$-check-F	ATTGTCGGCATACTTACGGG	
	$hdeD_{GI}$-check-R	ACAAGACGATAGCACCAAGG	
	orf11-check-F	GAAGCGATTGTCCGAGCTAAG	
	orf11-check-R	TGCTTGCCACTTCGTTATCC	
	trx_{GI}-check-F	ATCGGAACATCCCAACTGTG	
	trx_{GI}-check-R	CAGCCTCGGTATTCACCTTC	
	kefB-check-F	TTGCTGGGGTATCTCTCTGT	
	kefB-check-R	CAGCCACATCAATAGCAGGA	
	$pseiE_{GI}$-check-F	GTTTTTACGAGCGCTTCGAG	
	$pseiE_{GI}$-check-R	GAATGCTTGAACTCCATCGC	
	orf15-check-F	GCTAATGAGGATCTGCGTGT	
	orf15-check-R	GGCGATACGGCCAATAACAA	
	orf16-check-F	CGGAATCAATACCGCCATCT	

	orf16-check-R	GTCACGCGCAATACGAATAC	
Cm^R position	Cm-Insert	ATCCCTGGGTGAGTTTCACCAG	58
Complementation	*sHsp20*-NotI	GCGGCCGCAGTAAATGGACATCGATTTCAAGAA	60
	sHsp20-HindIII	AAGCTT TCAGCCGTTGATCGGGATCG	
	clpK$_{GI}$-NotI	GCGGCCGCAGTAAATGGCCAGAAAACAATGCCA	
	clpK$_{GI}$-HindIII	AAGCTTTCAAGATGCGTCGCTCGCCG	
	sHsp$_{GI}$-NotI	GCGGCCGCAGTAAATGTCTGCATTGACTCCGTG	
	sHsp$_{GI}$-HindIII	AAGCTTTTAGTTGACTGAGATTTCAA	
	pscA$_{GI}$ -NotI	GCGGCCGCAGTAAATGAATATCAAACAGCCAC	
	pscA$_{GI}$ -HindIII	AAGCTTTCAAAACAGCTTTTGGATGC	
	pscB-NotI	GCGGCCGCAGTAAATGAATGAGCAAACACCGAA	
	pscB -HindIII	AAGCTTTCAGAACAACCTGGACAGCT	
	hdeD$_{GI}$-NotI	GCGGCCGCAGTAAATGAATACAGACACCATCAC	
	hdeD$_{GI}$-HindIII	AAGCTTTTACCCCAGGCGCGTTTTGC	
	kefB-NotI	GCGGCCGCAGTAAATGCAGGGCTTGCTCGGCAC	
	kefB-HindIII	AAGCTTTCATGACGATGAACTCCCTG	
ddPCR	tLST-F	AGGTCTATTGGCCTGGTCTA	60
	tLST-R	AGCGGATTCCGGCAAAAA	60
	tLST-probe	/56-FAM/TGGCGTCCG/ZEN/TGCTGGCAAAA/3IABkFQ/	63
	WT-F	CCAGGCAAAGCGCCATTC	60
	WT-R	CTTTATGCTTCCGGCTCGTA	60
	WT-probe	/5HEX/GGCCTCTTC/ZEN/GCTATTACGCC /3IABkFQ/	63
