# Supplementary material for: Ecology and Function of the Transmissible Locus of Stress Tolerance in Escherichia coli and Plant-Associated Enterobacteriaceae
Source: mSystems. 2021 Aug 17;6(4):e00378-21. doi: 10.1128/mSystems.00378-21 (PMC8407380; doi:10.1128/mSystems.00378-21)
Supplement: TABLE S5 [file msystems.00378-21-st005.pdf]

Table S5. *E. coli* mutants used in this study.

Strain	Plasmids used in this strain	
	Complementation plasmid Chloramphenicol (34 mg/L)	Probe plasmid Ampicillin (100 mg/L)
E. coli MG1655		pCC_roGFP2_Orp1
E. coli MG1655 lacZ::LHR	pCA24N	pCC_roGFP2_Orp1
E. coli MG1655 lacZ::LHR Δ*orf1*		pCC_roGFP2_Orp1
E. coli MG1655 lacZ::LHR Δ*sHsp20*	pCA-*sHsp20*	pCC_roGFP2_Orp1
E. coli MG1655 lacZ::LHR Δ$clpK_{GI}$	pCA-$clpK_{GI}$	pCC_roGFP2_Orp1
E. coli MG1655 lacZ:: LHR Δ$sHsp_{GI}$	pCA-$sHsp_{GI}$	pCC_roGFP2_Orp1
E. coli MG1655 lacZ::LHR Δ$pscA_{GI}$	pCA-$pscA_{GI}$	pCC_roGFP2_Orp1
E. coli MG1655 lacZ::LHR Δ*pscB*	pCA-*pscB*	pCC_roGFP2_Orp1
E. coli MG1655 lacZ::LHR Δ$hdeD_{GI}$	pCA-$hdeD_{GI}$	pCC_roGFP2_Orp1
E. coli MG1655 lacZ::LHR Δ*orf11*		pCC_roGFP2_Orp1
E. coli MG1655 lacZ::LHR Δtrx_{GI}		pCC_roGFP2_Orp1
E. coli MG1655 lacZ::LHR Δ*kefB*	pCA-*kefB*	pCC_roGFP2_Orp1
E. coli MG1655 lacZ::LHR Δ$pseiE_{GI}$		pCC_roGFP2_Orp1
E. coli MG1655 lacZ::LHR Δ*orf15*		pCC_roGFP2_Orp1
E. coli MG1655 lacZ::LHR Δ*orf16*		pCC_roGFP2_Orp1
